# Complete genome analysis of the African swine fever virus genotypes II and IX responsible for the 2021 and 2023 outbreaks in Rwanda

**DOI:** 10.3389/fvets.2025.1532683

**Published:** 2025-02-18

**Authors:** Jean Nepomuscene Hakizimana, Clara Yona, Mariam Richard Makange, Ester Kasisi Adamson, Pie Ntampaka, Evodie Uwibambe, Method Ngabo Gasana, Fabrice Ndayisenga, Hans Nauwynck, Gerald Misinzo

**Affiliations:** ^1^OR Tambo Africa Research Chair for Viral Epidemics, SACIDS Foundation for One Health, Sokoine University of Agriculture, Morogoro, Tanzania; ^2^Department of Biosciences, College of Natural and Applied Sciences, Sokoine University of Agriculture, Morogoro, Tanzania; ^3^Department of Veterinary Medicine, College of Agriculture, Animal Science and Veterinary Medicine, University of Rwanda, Nyagatare, Rwanda; ^4^Department of Microbiology, Parasitology and Biotechnology, Sokoine University of Agriculture, Morogoro, Tanzania; ^5^Rwanda Agriculture and Animal Resources Development Board, Butare, Rwanda; ^6^Laboratory of Virology, Faculty of Veterinary Medicine, Ghent University, Merelbeke, Belgium

**Keywords:** African swine fever virus, domestic pigs, genomics, genotype II, genotype IX, Rwanda

## Abstract

African swine fever (ASF) is a devastating viral hemorrhagic disease caused by the ASF virus (ASFV) that can kill up to 100% of domestic pigs and wild boars. The domestic pig industry in Rwanda is highly threatened by ASF, with several outbreaks reported yearly to the World Organization for Animal Health. Despite the endemic status, no ASFV from Rwanda has been genetically characterized. This study reports, for the first time, the ASFV genotypes causing outbreaks in Rwanda. The ASF confirmation was performed by polymerase chain reaction followed by molecular characterization of the causative ASFV by partial and complete genome sequencing and phylogenetic reconstruction. After genetic analysis, the ASFV strains responsible for the 2021 outbreak in eastern Rwanda clustered within genotype II, while the strain from the 2023 outbreak in northern Rwanda clustered within genotype IX. The extension of the geographical range of genotype II in eastern Africa is of concern. In the countries of the East African Community, this ASFV genotype was reported for the first time in Tanzania at the Tanzania-Malawi border in 2011, followed by a relentless spread of the virus northwards along major highways within Tanzania before the detection of this genotype in Rwanda in 2021. This ASFV genotype will most likely reach other eastern African countries threatening the regional domestic pig industry. The ongoing spread of ASFV genotypes II and IX across Africa impacts food and nutritional security, and hinders the realization of the United Nations Sustainable Development Goal 1 (No Poverty) and·Goal 2 (Zero hunger). The results of this study call for science-driven and regional approaches to enable the timely identification of ASF outbreaks for effective prevention and containment.

## Introduction

In Rwanda, animal health is the main area targeted for interventions to increase the livestock productivity in order to meet the rising demand for animal-source proteins (1). Domestic pig production contributes significantly to food security, improved nutrition and livelihoods of farmers with 1.8 million domestic pigs of which 80% are owned by smallholder domestic pig farmers in Rwanda (2). However, the domestic pig industry is highly threatened by African swine fever (ASF), a transboundary animal disease with up to 100% mortality rate in naïve populations of domestic pigs and Eurasian wild boars and has neither a world-scale commercially available vaccine nor treatment at the moment (3, 4). The disease is caused by the ASF virus (ASFV) a double-stranded DNA virus maintained in a sylvatic cycle involving soft ticks of the *Ornithodoros moubata* complex as vectors while the asymptomatically infected wild suids, mainly warthog (*Phacochoerus africanus*) play an important role as ASFV reservoirs (5, 6). Since its first description in 1921 in Kenya (7), the disease remained confined to certain countries of Africa South of the Sahara until 1957, when the disease spread to Europe, South America and the Caribbean (8). There are 24 (I-XXIV) genotypes of ASFV that have been described so far in Africa, and the ASFV genotype I is the one that escaped from Africa in 1957 (9, 10). In 2007, ASFV genotype II escaped from the African continent to Georgia, spread throughout the Caucasus and the Russian Federation, and in 2014 the infection reached the European Union (11, 12). In 2018, ASF was introduced to China followed by subsequent spread to many other countries in Asia (13, 14). In 2021, ASFV genotype II was reported in Haiti and the Dominican Republic of South America with devastating negative impact on the domestic pig industry (15–17). The ASF is now a disease of global concern, negatively impacting the global pork products trade, food and nutritional security, particularly due to the recent spread of the ASFV genotype II into previously unaffected countries (17–22). Recently, on the African continent, ASFV genotype II was reported for the first time in Nigeria (19, 23) and Ghana (21) underscoring the ongoing expansion of the geographical distribution of the current ASFV pandemic. Ghana and Nigeria are West African countries which had been previously dominated by the presence of ASFV genotype I only. The cocirculation of ASFV genotypes I and IX has been documented in the Republic of Congo (Congo-Brazzaville) indicating the geographical expansion of genotype IX from its original confinement in eastern Africa to central Africa increasing the threat of continuing spread of this ASFV genotype (24).

In eastern and southern Africa, since the first description of ASF in 1921 in Kenya, the disease has become endemic among domestic pig population representing an important constraint for the development of the domestic pig industry (25). The existence of favoring conditions including the dominance of a traditional extensive family-based production system characterized by inadequate biosecurity system represents a major challenge for the prevention and control of ASF (2, 26, 27). In addition, the existence of a poorly understood warthog-tick sylvatic cycle of ASF in eastern and southern Africa, a region that hosts a large diversity of ASFV genotypes combined with a low level of surveillance represents a constant threat of recursive introductions of new ASF genotypes into the domestic pig value chain (5, 6, 28). The eradication of ASF is hindered by the existence of ASFV sylvatic cycle and the most feasible approach is to focus on preventing the transmission of the virus from the sylvatic cycle to domestic pig population and application of effective evidence-based control strategy in case of ASFV introduction into the domestic pig population (6).

Several ASF outbreaks have been reported to the World Organization for Animal Health (WOAH) by veterinary authorities of Rwanda. For instance, from 2005 to 2019, Rwanda reported to WOAH a total of 381 ASF outbreaks affecting 11,203 domestic pig cases leading to 7,463 deaths (29, 30). Despite the endemic status, no ASFV from Rwanda has been genetically characterized. The availability of ASFV complete genomes provides a baseline for research on the development of effective control and prevention strategies, including vaccines, diagnostic tests and antiviral treatment development. The objective of this study was to describe the ASFV genotypes responsible for the 2021 and 2023 outbreaks in Rwanda using partial and complete genome sequencing for a better understanding of the transboundary spread of ASFV and possible control strategies.

## Materials and methods

### Samples description

**Figure 1:**
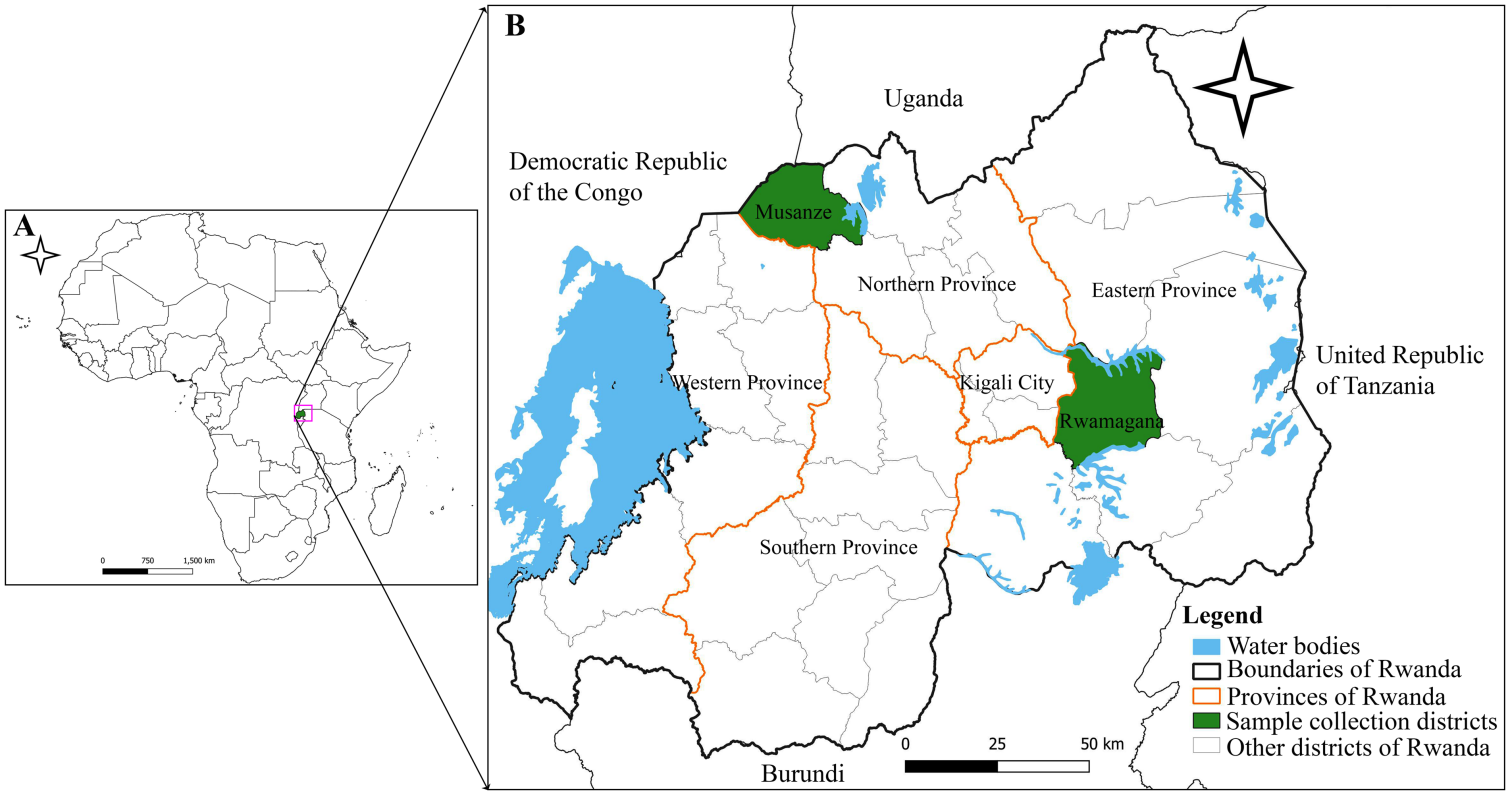
Map showing the location of the African swine fever outbreaks in Rwamagana district, eastern Rwanda in 2021 and in Musanze district, northern Rwanda in 2023. **(A)** Map of Africa showing the location of Rwanda (green). **(B)** Map of Rwanda showing Rwamagana and Musanze districts where samples used in the present study were collected. The map was developed by authors using QGIS software version 3.24.1 and data from DIVA-GIS freely available at https://www.qgis.org/en/site/ and https://diva-gis.org/, respectively.

Deadly hemorrhagic fever outbreaks were reported in domestic pigs in Rwanda during 2021 and 2023, affecting the districts of Rwamagana and Musanze, respectively. In Rwamagana, the outbreak occurred in the Muyumbu sector in September 2021 resulting in the death of 32 domestic pigs, while in Musanze, the outbreak was reported in the Muko sector in February 2023 with 200 domestic pig deaths recorded. The data on pig mortality during these outbreaks was obtained from the records of the Rwanda Agriculture and Animal Resources Development Board (RAB). Four (4) tissue samples comprising liver and spleen aseptically collected from domestic pigs dead from suspected ASF outbreak in Rwamagana district, eastern Rwanda were used for diagnosis and molecular characterization of the ASFV responsible for the 2021 outbreak in Rwanda (Figure 1). In addition, five tissue samples collected in Musanze district, northern Rwanda from domestic pigs dead from suspected ASF outbreak were used in this study to describe the ASFV that caused the 2023 outbreak.

### Confirmation of ASFV and genotype assignment

In the laboratory, viral DNA was extracted from collected domestic pig samples using the QIAamp DNA purification kit (Qiagen, Hilden, Germany), following the protocol provided by the manufacturer. The extracted DNA was used for ASF diagnosis using polymerase chain reaction using p72D and p72U primers on the Eppendorf Mastercycler nexus PCR thermal cycler (Eppendorf, Hamburg, Germany) as previously described (31). The size of the generated PCR products was verified using 1.5% agarose gel electrophoresis and visualized on BioDoc-It imaging system (Bio-Rad, Hercules, CA) followed by dideoxynucleotide cycle sequencing using an ABI 3730xl DNA analyzer (Applied Biosystems, Foster City, CA). The sequencing of the PCR products was outsourced from Macrogen (Macrogen Europe, Amsterdam, The Netherlands) and the generated sequences were assembled and used for the classification of ASFV strains described in this study among the already described 24 ASFV genotypes.

### Sequencing library preparation and next-generation sequencing

The next-generation sequencing libraries were prepared using the Illumina DNA Prep kit (Catalog # 20018704, Illumina, CA, United States), following manufacturer’s instructions. The resulting libraries were pooled, normalized, and quantified using the Qubit DNA High Sensitivity kit (Thermo Fisher Scientific, Waltham, United States) according to the manufacturer’s recommended protocol. Paired-end sequencing was performed in-house using an iSeq 100 (Illumina, CA, United States) with 300-cyle iSeq 100 i1 Reagents v2 (Catalog # 20031371, Illumina, CA, United States) and sequencing reads were generated with a configuration of 2 × 151 base pairs.

### Bioinformatics analysis

The generated Binary Base Call (BCL) files were converted to Fastq files using bcl2fastq version 2.19.0.316 (Illumina, CA, United States). To gather information regarding the overall quality of the reads, including total bases, total reads, GC content, and basic statistics, the raw sequencing reads were subjected to quality control using FastQC version 0.11.9 (32). Trimming of sequencing adapters and low-quality ends from reads was performed using trim_galore version 0.6.4 powered by cutadapt version 4.5.^1^ The quality Phred score cutoff was set at 30 with a minimum read length of 75 base pairs (bp). The quality-filtered sequencing reads were then mapped to Georgia2007/1 (GenBank accession number FR682468.2) and Ken06.bus (GenBank accession number NC_044946.1) ASFV reference genomes for genotypes II and IX, respectively. The mapped sequencing reads were *de novo* assembled using SPAdes genome assembler version 3.13.1 (33) and the quality of the resulting assemblies was evaluated using the Quality Assessment Tool (QUAST) program version 5.0.2 (34). The assembled genomes were annotated using the Genome Annotation Transfer Utility (GATU) program (35).

For phylodynamic analysis, p72 ASFV nucleotide sequences obtained in this study along with those previously reported downloaded from the National Center for Biotechnology Information (NCBI) GenBank were aligned using Mafft version 7. 453 (36). The alignment consisted of 102 nucleotide sequences collected between 1954 and 2023 including p72 ASFV nucleotide sequences from Burundi (*n* = 3), Democratic Republic of the Congo (*n* = 12), India (*n* = 1), Kenya (*n* = 15), Malawi (*n* = 9), Malaysia (*n* = 1), Mozambique (*n* = 11), Nigeria (*n* = 1), Rwanda (*n* = 4), Tanzania (*n* = 20), Uganda (*n* = 12), Vietnam (*n* = 1) and Zambia (*n* = 12). The best-fitting model was identified using ModelFinder implemented in IQ-TREE version 1.6.12 for Linux (37) and the Kimura 2-parameter model with gamma distribution (K2P + G4) was selected based on the Bayesian Information Criterion (BIC). A maximum likelihood phylogenetic tree was reconstructed using IQ-TREE version 1.6.12 and tempest version 1.5.3 was used to investigate the temporal signal and clock likeness of the used dataset (38). The strict molecular clock model was used to infer the divergence times for the ASFV p72 nucleotide sequences using the Bayesian Evolutionary Analysis Utility (BEAUti) version 1.10.4 and the Bayesian Evolutionary Analysis Sampling Trees (BEAST) version 1.10.4 (39). The posterior probability density was estimated using the Markov Chain Monte Carlo (MCMC) with a chain length of 15 × 10^6^ sampling after every 1,000 cycles. The results generated by BEAST were explored using Tracer version 1.7.2 targeting an effective sample size of at least 200 for each parameter (40) followed by the generation of the target tree using TreeAnnotator version 1.10.4 after a burn-in of 20% of the sample and visualization by FigTree version 1.4.4 (41). In addition, Maximum Likelihood phylogenetic tree was reconstructed using the ASFV whole genome nucleotide sequences described in this study along with those previously described retrieved from the NCBI GenBank nucleotide database.

## Results

### Genotype assignment and general features of the complete genomes of ASFV responsible for outbreaks in Rwanda in 2021 and 2023

Outbreaks of deadly hemorrhagic fever were reported in domestic pigs in 2021 and 2023 in Rwamagana and Musanze districts of Rwanda, respectively. Tissue samples were collected from dead domestic pigs and after laboratory molecular detection targeting the *B646L* gene encoding for the p72 major ASFV capsid protein, the collected samples were ASFV-positive. After genetic analysis using partial and complete genome nucleotide sequences, the ASFV strains responsible for the 2021 outbreak in Rwamagana district in eastern Rwanda clustered within genotype II, while the strain from the 2023 outbreak in Musanze district in northern Rwanda clustered within genotype IX. This study describes two complete genome sequences of ASFV strains from the 2021 and 2023 outbreaks in Rwanda designated as ASFV/RWA/Rwamagana/2021 and ASFV/RWA/Musanze/2023. The assembled ASFV strains had a genome size of 183,853 and 184,517 base pairs (bp) with an average GC content of 38.45 and 38.54% for the strains ASFV/RWA/2021/Rwamagana and ASFV/RWA/2023/Musanze, respectively (Table 1). After genome annotation, the ASFV/RWA/2021/Rwamagana strain contained 183 open reading frames (ORFs) while the ASFV/RWA/2023/Musanze strain had 160 ORFs.

**Table 1:** Basic statistics of the sequencing results of the African swine fever virus complete genome sequences responsible for outbreaks in Rwanda in 2021 and 2023.

Isolate	Total number of reads	#ASFV specific reads	Percentage of ASFV specific reads (%)	Mean bases phred quality score	Assembled ASFV genome size (bp)	Mean genome coverage depth	GC content (%)	GenBank accession number	p72 genotype
ASFV/RWA/Rwamagana/2021	33,852,278	73,721	0.22	35.7	183,853	56.8x	38.45	PQ375363	II
ASFV/RWA/Musanze/2023	8,359,266	60,444	0.72	35.4	184,517	48x	38.54	PQ375362	IX

### Phylogenetic and comparative genomics analysis

**Figure 2:**
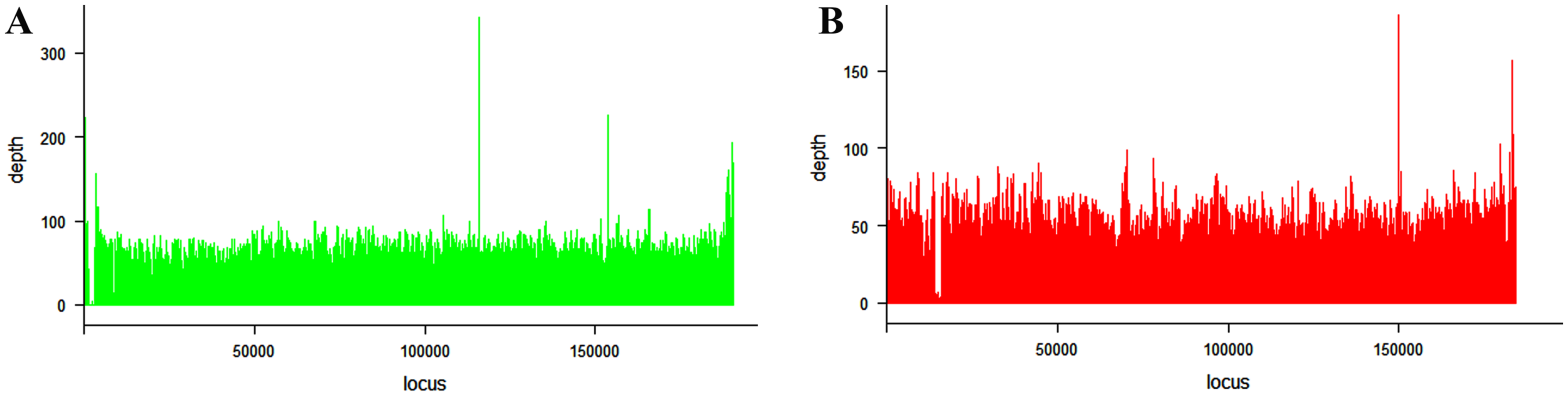
Coverage plots for the African swine fever virus genotype II **(A)** collected from Rwamagana district in eastern Rwanda in 2021 and genotype IX **(B)** collected from Musanze district in northern Rwanda in 2023. The coverage was calculated using samtools and plotted using lattice package in RStudio.

The ASFV complete genome sequences described in this study were compared to those previously characterized and available at the NCBI GenBank database. The strain ASFV/RWA/Rwamagana/2021 was closely related to ASFV genotype II collected from Rukwa region in 2017 in southwestern Tanzania with 99.97% nucleotide identity and 99% query coverage. In addition, a nucleotide identity of over 99.90% was observed with other ASFV genotype II isolates reported from different countries in Africa, Europe and Asia. On the other hand, the strain ASFV/RWA/Musanze/2023 exhibited over 99.80% nucleotide identity to ASFV genotype IX isolates previously described from Uganda and Kenya. When compared to ASFV reference genomes, the mean genome coverage depths were 56.8 and 48x for ASFV/RWA/Rwamagana/2021 and ASFV/RWA/Musanze/2023 ASFV strains, respectively (Figure 2).

**Figure 3:**
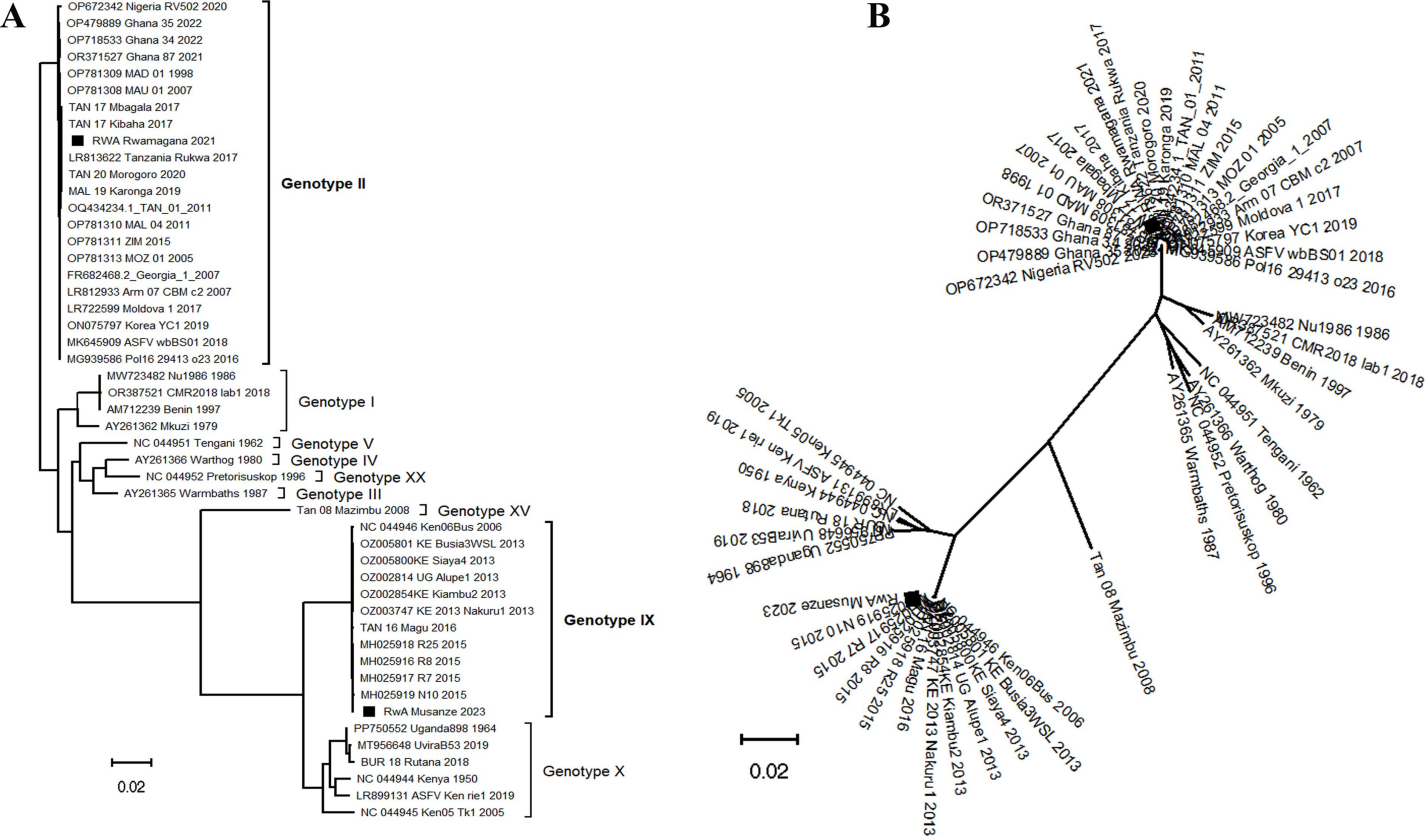
Phylogenetic tree reconstructed using African swine fever virus (ASFV) complete genomes nucleotide sequences collected from Rwanda in 2021 and 2023 along with sequences previously described available at the NCBI GenBank. **(A)** The traditional rectangular phylogenetic tree with the ASFV strains described in this study indicated by a black square. **(B)** The radiation format of the phylogenetic tree showing six distinct clusters of the available ASFV complete genomes. The scale bar indicates nucleotide substitution per site.

After Maximum Likelihood phylogenetic tree reconstruction using ASFV complete genome nucleotide sequences, the strains described in this study clustered within genotypes II and IX for ASFV/RWA/Rwamagana/2021 and ASFV/RWA/Musanze/2023 strains, respectively (Figure 3A). The radiation format of the reconstructed phylogenetic tree showed six distinct clusters of the analyzed database of ASFV complete genomes (Figure 3B).

### Phylodynamic analysis

**Figure 4:**
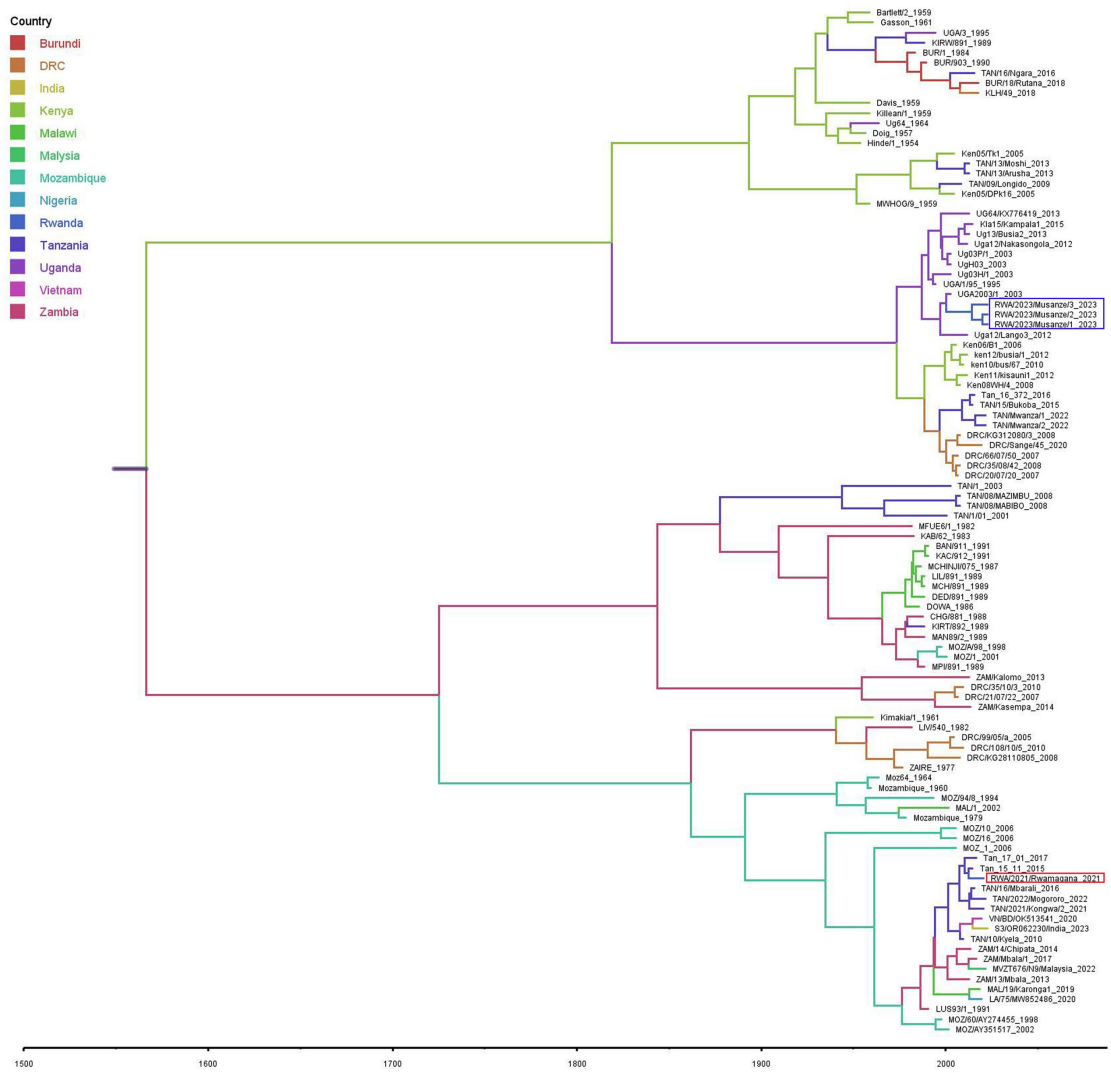
Maximum clade credibility phylogenetic tree obtained using the African swine fever virus *B646L* (p72) gene nucleotide sequences including those described in this study collected from Rwanda in 2021 and 2023 (in red and purple rectangles, respectively) and those previously characterized available at the NCBI GenBank. Branches on the tree are colored according to the most likely location of the descendant, and the scale axis represents calendar years.

The divergence-time analysis showed that the time to the most recent common ancestor (TMRCA) for the analyzed p72 ASFV nucleotide sequence dataset was 1,541 with 95% highest posterior density (HPD) interval ranging from 1,268 to 1760. The estimated nucleotide substitution rate was 7.6609 × 10^−5^ substitutions/site/year with a 95% HPD interval ranging from 3.8017 × 10^−5^ to 1.1483 × 10^−4^. The phylodynamic analysis indicated that the ASFV responsible for the 2021 outbreak in Rwamagana district was closely related to isolates previously described in Tanzania while on the other hand, the ASFV responsible for the 2023 outbreak in Musanze district clustered closely with isolates previously reported from Uganda (Figure 4).

## Discussion

The ASF constitutes a major threat to the global domestic pig industry, international trade market, food and nutritional security. Since outbreaks of ASF can spread rapidly across a country, regions and, in some cases, become global, it is important to understand the origin and transmission dynamics of responsible ASFV strains in order to develop and apply effective containment strategies. Although partial genome characterization is a crucial step that allows classification of ASFV strain among the known genotypes, whole genome sequencing provides the most detailed and comprehensive insights into the viral transmission dynamics during outbreaks. In this study, partial and complete genome sequencing were used to characterize the ASFV strains responsible for outbreaks in 2021 and 2023 in Rwanda. Genetic analysis revealed that the ASFV strains responsible for the 2021 outbreak in Rwamagana district in eastern Rwanda belonged to genotype II, while the strain from the 2023 outbreak in Musanze district in northern Rwanda clustered within genotype IX. In addition, phylodynamic analysis indicated that the ASFV responsible for the 2021 outbreak in Rwamagana district was closely related to isolates previously described in Tanzania while on the other hand, the ASFV responsible for the 2023 outbreak in Musanze district clustered closely with isolates previously reported from Uganda. After phylogenetic tree reconstruction using ASFV complete genome nucleotide sequences, six distinct clusters were identified, with genotypes IX and X forming two distinct clusters as previously reported (42).

The high genetic similarity between ASFV genotype II responsible for the outbreak in Rwanda in 2021 and other isolates previously reported in Africa, Europe and Asia highlight the widespread of this ASFV genotype responsible for the current global pandemic. In eastern and southern Africa, the genotype II of ASFV has been previously reported in Malawi (43), Mozambique (9), Tanzania (44, 45), Zambia (46) and Zimbabwe (47). The eastern and southern African region or Madagascar has been reported to be the most likely origin of the ASF incursion in Georgia in 2007 followed by global spread with devastating negative impact on domestic pig industry (11). Of concern is the extension of the geographical distribution of genotype II across western and eastern Africa (19, 21, 48). In the countries of the East African Community, this genotype was first reported in Tanzania at the Tanzania-Malawi border in 2011, followed by a relentless spread of the virus northwards along major highways within Tanzania before the detection of this genotype in Rwanda in 2021 (44, 45). Uncontrolled domestic pig movements and low level of biosecurity have been cited as the main drivers of ASF spread in Tanzania (27, 49). In this study, nucleotide sequence identity of 99.97% between the ASFV responsible for outbreak in Rwamagana district in eastern Rwanda and ASFV genotype II collected in South-western Tanzania in 2017 was observed suggesting a possible cross-border transmission of ASF between the two countries. In fact, Tanzania and Rwanda share a common border and maintain active trade relations, including the exchange of agricultural products and livestock with a major highway connecting both countries. The uncontrolled movement of pigs and pork products across the border, coupled with the frequent trade activity, increases the risk of cross-border transmission of ASF. A high number of ASF outbreaks in the vicinity of major highway due to illegal transportation of infected domestic pigs and pork products has been documented in Tanzania (45). The possible transboundary spread of ASF between Rwanda and Tanzania was inferred after phylogeographic analysis using the Bayesian approach. These findings are in agreement with previous studies that identified the possibility of transboundary spread of ASF in eastern and southern Africa (30, 50). This ASFV genotype will most likely reach other eastern African countries threatening the regional domestic pig industry if appropriate control measures are not applied.

The outbreak of ASF in Musanze district in northern Rwanda in 2023 was caused by ASFV genotype IX. This genotype has been described as the most prevalent in eastern Africa and it has been previously reported in Democratic Republic of the Congo (51), Kenya (50), Tanzania (52) and Uganda (53). A nucleotide identity of over 99.80% between the ASFV genotype IX described in this study and strains previously reported in different countries of eastern Africa has been observed in this study suggesting a possible common origin followed by subsequent spread in the region. Phylodynamic analysis showed that Uganda is the most likely source of the 2023 ASFV strain detected in Musanze district in northern Rwanda. These findings are consistent with previous studies that have identified the existence of a domestic pig associated genotype IX responsible for outbreaks in eastern Africa with transboundary transmission between countries (30, 50, 53). Musanze district, where the 2023 ASF outbreak occurred, is situated along the Rwanda-Uganda border where illegal movements of domestic pigs and pork products are likely to occur. However, considering previously reported ASF outbreaks in Rwanda without molecular characterization of the causative ASFV strains and the existence of viral wild hosts in different wildlife protected areas in the country, the re-emergence of an in-country strain cannot be excluded. Further investigations are recommended for an improved understanding of the epidemiology and transmission dynamics of ASFV in Rwanda focusing mainly on the wildlife-domestic pigs’ interface. The ongoing spread of ASFV genotype IX across Africa poses a risk of spreading beyond the continent and potentially impacting the domestic pig industry globally.

The estimated TMRCA dated back to 1,541 (95% HPD: 1268–1760) supporting the hypothesis that ASFV was silently circulating most likely in wildlife reservoirs hosts in eastern Africa before its emergence in domestic pigs population as previously reported (30, 54, 55). The evolution rate was 7.6609 × 10^−5^ (95% HPD: 3.8017 × 10^−5^-1.1483 × 10^−4^) substitutions per site per year highlighting a rapid evolutionary dynamic of ASFV as compared to its counterpart double-stranded DNA viruses as previously documented (56). The findings of this study are consistent with those of previous studies that analyzed the genomics evolution of ASFV elsewhere (30, 54, 55, 57). The ASFV substitution rate which is within the range of that observed in single-stranded DNA viruses (58–60) may explain the high genetic diversity observed within ASFV strains resulting in negative impact on vaccine development and disease surveillance (61).

In conclusion, this study demonstrated that the ASFV strains responsible for outbreaks in Rwamagana and Musanze districts in Rwanda belonged to genotypes II and IX, respectively. Phylogeographic and phylogenetic analyses revealed potential inter-countries viral spread events and a high genetic similarity between ASFV strains from Rwanda and those previously described from neighboring countries suggesting a possible transboundary transmission of the ASF between different countries in eastern Africa. Continued genomics surveillance of ASFV in domestic pigs and wild host reservoirs in wildlife protected areas in Rwanda is essential to fully understand the transmission dynamics of the disease in the country. In this context, a science-driven regional approach is recommended to ensure timely detection of ASF outbreaks and the application of effective prevention and containment strategies including contextualized biosecurity measures and strict movement controls.

## Data Availability

The datasets presented in this study can be found in online repositories. The names of the repository/repositories and accession number(s) can be found at: https://www.ncbi.nlm.nih.gov/genbank/, PQ375363 and PQ375362.

## Footnotes

^1^ https://www.bioinformatics.babraham.ac.uk/projects/trim_galore/
